# The PARP inhibitor olaparib promotes senescence in murine macrophages

**DOI:** 10.1007/s11357-025-01679-6

**Published:** 2025-05-06

**Authors:** Anna Kieronska-Rudek, Karim Zuhra, Kelly Ascenção, Stefan Chlopicki, Csaba Szabo

**Affiliations:** 1 Section of Pharmacology, Department of Oncology, Microbiology and Immunology, Faculty of Science and Medicine, University of Fribourg, Fribourg, Switzerland; 2 Jagiellonian Centre for Experimental Therapeutics, Jagiellonian University, Krakow, Poland

**Keywords:** PARPs, Olaparib, Senescence, Macrophages, Aging

## Abstract

Cellular senescence is a multifaceted process involving cell cycle arrest, telomere shortening, and the accumulation of DNA damage associated with aging and cellular stress. It is marked by persistent cell cycle arrest and DNA damage accumulation, and plays an increasingly recognized role in age-related diseases and cancer therapy. Olaparib, a poly (ADP-ribose) polymerase (PARP) inhibitor, is approved for use in ovarian cancer treatment. We hypothesized that olaparib may influence senescence by inhibiting DNA damage repair, and investigated its effects on non-senescent and replicatively senescent murine macrophages (RAW 264.7 cells). Senescent cells exhibited elevated baseline levels of PARP1 expression, PARylation, and DNA damage relative to non-senescent control cells. Olaparib amplified these differences by upregulating senescence markers (SA-β-gal and p21), inhibiting proliferation, and exacerbating DNA damage. Many of its effects were more pronounced in senescent cells. At higher concentrations (10–30 µM), olaparib induced significant cytotoxicity through mixed apoptotic and necrotic mechanisms, with senescent cells exhibiting a predominantly necrotic response. Interestingly, both mitochondrial activity and cellular bioenergetics were elevated in senescent cells at baseline, and were more severely impaired by olaparib compared to non-senescent control cells. These findings underscore olaparib’s enhanced cytotoxic and pro-senescent effects in senescent immune cells and suggest potential implications for its use in elderly cancer patients with an increased burden of senescent cells.

## Introduction

Poly(ADP-ribose) polymerases (PARPs) constitute a family of enzymes that catalyze the transfer of ADP-ribose units to target proteins—a post-translational modification known as PARylation. This post-translational modification plays a critical role in regulating a variety of cellular processes, such as chromatin remodeling, transcription, replication, recombination, and DNA repair [1–4]. The principal member of this family is PARP1, a constitutive nuclear and mitochondrial protein. PARPs are integral to maintaining cellular homeostasis and responding to DNA damage. PARP inhibitors are used in cancer therapy, but PARP activation also plays a role in various pathophysiological conditions, including cardiovascular, inflammatory and neurological diseases [1–4].

Olaparib, marketed under the commercial name Lynparza®, is a PARP inhibitor approved as a first-line maintenance treatment for several cancers, including ovarian and certain breast cancer [4, 5].

Although the link between PARPs and senescence has been studied for decades [reviewed in 6–14], the available information on the effect of olaparib on cellular senescence is relatively limited, and focuses on cancer cells, as opposed to senescent non-transformed mammalian cells [15–19]. How olaparib influences senescent cells –– especially non-transformed parenchymal and immune cells –– remains to be further investigated. In the present study, we examined the differential effects of olaparib on senescent and non-senescent cells using an in vitro murine macrophage model.

## Materials and methods

### Materials

Olaparib was purchased from MedChemExpress (Lucerne, Switzerland). The Comet assay kit (cat. 4250-050-K) was purchased from Biotechne (Zug, Switzerland). The cell proliferation ELISA, BRDU (colorimetric) kits were purchased from Roche (Basel, Switzerland). SYBR Gold was purchased from Thermo Fisher Scientific (Waltham, MA, USA). Antibodies used in the present work were purchased as follows: poly-ADP-ribose (PAR; ALX-804-220-R100, Enzo, Lausen, Switzerland), PARP1 (sc-74470, Santa-Cruz, Heidelberg, Germany), β-actin (A1978, Sigma Aldrich), Annexin V (640920, Sigma Aldrich), while propidium iodide (PI; 421301; BioLegend, San Diego, CA, USA), p21 (28248-1-AP), phospho-histone H2 A.X (Ser139; 80312) and X-ray cross-complementing protein 1 (XRCC1; 76998) were purchased from Proteintech (Manchester, UK).

### Cell culture, generation of replicative senescent cells

RAW 264.7 cells (a murine macrophage cell line) were purchased from the American Type Culture Collection (ATCC TIB-71). Cells were cultured in Dulbecco’s Modified Eagle’s Medium DMEM (Gibco, Thermo Fisher Scientific, Waltham, MA, USA) supplemented with 10% fetal bovine serum (Gibco) at 37 °C in a humidified atmosphere containing 5% CO_2_. Non-senescent and senescent cells used in the experiments were at 5–10 and 30–40 passages, respectively. The various markers of replicative senescence in RAW 264.7 cells have been characterized previously [20].

### Immunocytochemical staining

The expression of p21 was also analyzed in non-senescent vs. senescent RAW 264.7 cells treated with olaparib (0, 1, 3, 10, and 30 µM) for 72 h. In addition, the expression of phospho-histone H2 A.X (Ser139) and XRCC1 was analyzed to investigate DNA damage in olaparib-treated cells. Immunocytochemical staining was performed according to a previously described protocol [20]. Briefly, cells were fixed in 4% PFA for 10 min, then incubated for 30 min with a solution containing 2% non-fat dry milk and 5% goat serum (Jackson Immuno Research, Cambridgeshire, UK) and 0.05% Triton X-100 to permeabilize the membranes and prevent non-specific antibody binding. After three washes with DPBS, cells were incubated overnight with primary antibodies against p21 (1:200), phospho-histone H2 A.X (Ser139; 1:200), or XRCC1 (1:100), followed by incubation with appropriate secondary antibodies AlexaFluor 488-conjugated goat anti-mouse or AlexaFluor 568-conjugated goat anti-rabbit (Thermo Fisher Scientific). Nuclei were stained with DAPI (1:2,000) for 10 min. Subsequently, 4 (at 100× magnification) or 9 pictures (at 200× magnification) per well were collected using the Citation5 system. Quantitative analysis was performed using Gen5 software. Additionally, representative images of phospho-histone H2 A.X (Ser139) and XRCC1 were captured using a Leica TCS SP5 Confocal microscope.

### Western blotting

Cell lysates were prepared using RIPA Lysis and Extraction Buffer (Thermo Fisher Scientific) supplemented with Halt™ Protease and Phosphatase Inhibitor Cocktail (Thermo Fisher Scientific). Protein content in lysates and homogenates was analysed using the Pierce BCA Protein Assay Kit according to the manufacturer’s instructions (Thermo Fisher Scientific). Western blot analysis was performed as described [21]. Briefly, samples were prepared using 1x Invitrogen Bolt^TM^ LDS sample buffer (Thermo Fisher Scientific) and 1x Invitrogen Bolt^TM^ Sample Reducing Agent (Thermo Fisher Scientific). Electrophoresis was performed using an Invitrogen Western Blot electrophoresis system using Invitrogen Bolt^TM^ Bis-Tris gradient protein gels. Subsequently, proteins were transferred onto Invitrogen iBlot PVDF membranes. Blocking was achieved with the 5% nonfat dry milk. The following primary antibodies were used: PAR (1:2000), PARP1 (1:1000), and β-actin (1:15,000). The membranes were incubated with appropriate horseradish peroxidase (HRP)-conjugated secondary antibodies (Cell Signaling Technology, Danvers, MA, USA). The blots were developed using Radiance Plus Chemiluminescence solution (AC2103, Azure Biosystem Dublin, CA, USA). Densitometry analysis was performed using the ImageJ software (National Institutes of Health, Bethesda, MD, USA). The values of individual proteins were normalized to the expression of β-actin.

### Measurement of senescence-associated ß galactosidase activity

To assess SA-β-galactosidase activity, cells were seeded in 6-well plates at a density of 5 × 10^5^ cells per well and treated with olaparib (0, 1, 3, 10, or 30 µM) for 72 h. The activity of SA-β-gal was determined using a commercial kit (Sigma-Aldrich, Switzerland) according to the manufacturer’s protocol. Staining was performed overnight in a humidified 37 °C incubator without CO_₂_. After washing, cells were visualized using a bright-field optical microscope (Olympus CKX53, Tokyo, Japan). Quantitative analysis was conducted in ImageJ using pre-segmented images (Ilastik 1.3.0 software; developed by the Ilastik team, with partial financial support by the Heidelberg Collaboratory for Image Processing, HHMI Janelia Farm Research Campus and CellNetworks Excellence Cluster) as described in detail [22, 23]. Data are expressed as a ratio of SA-β-gal positive area over total cell area.

### Measurement of cell proliferation

Cells were seeded in 96-well plates at a density of 1.25 × 10^3^ and incubated for 72 h with 1, 3, 10, 30 μM olaparib or vehicle. Cell proliferation was assessed using a colorimetric BrdU ELISA kit (Roche). Cells were incubated with 10 µM BrdU labelling solution for 1 h at 37 °C and 5% CO_2_. Subsequently, cells were fixed for 25 min using the fixation buffer provided with the kit. Fixed cells were incubated for 1 h with an anti-BrdU antibody, washed 3 times in DPBS, and incubated for 5 min with the colorimetric substrate. Absorbance was measured at 450 nm with 690 nm as the reference wavelength using a Spectramax plate reader (Molecular Devices, San Jose, CA, USA).

### Measurement of cell viability

Cells were seeded in 96-well plates at a density of 2.5 × 10^3^ and incubated for 72 h with olaparib (0, 1, 3, 10, 30 μM) or vehicle. For the measurement of lactate dehydrogenase (LDH) activity, 50 µl of each well’s supernatant was transferred into a new plate. The remaining medium was discarded, cells were lysed using RIPA buffer, total protein was quantified, and results were normalized to cellular protein content. The LDH assay was performed as described [24] using the Pierce LDH Cytotoxicity Assay Kit (Thermo Scientific). Briefly, the LDH mixture was prepared according to the manufacturer’s instructions, and 50 µl/well was added to the supernatants. The plate was then incubated for 30 min at room temperature protected from light, and the reaction was stopped with 25 µl/well of Stop Solution provided with the kit. Absorbance was measured after 60 min of shaking, using an Infinite 200 Pro reader (Tecan) at 490 nm and reference wavelength 680 nm for background.

The MTT assay was performed as described [24]. The remaining medium was discarded and 100 µl/well of 3-(4,5-dimethylthiazol-2-yl)−2,5-diphenyltetrazolium bromide (MTT, 0.5 mg/mL) was added to the cells for 1-h incubation (37 °C and 5% CO_2_), to test mitochondrial activity. Subsequently formazan crystals were dissolved in 100 µl DMSO with orbital shaking for 10 min in the dark. The absorbance was measured at 590 nm using an Infinite 200 Pro reader (Tecan).

ATP levels were measured using the CellTiter-Glo® Luminescent Cell Viability Assay (Promega, Madison, WI, USA) according to the manufacturer’s instructions. A standard ATP curve was prepared by serially diluting ATP to final concentrations of 1 µM, 100 nM, 10 nM, 1 nM, and 0 nM in culture medium. These standards (100 µL per well) were loaded into the empty wells of a 96-well plate, which matched the volume of the medium in which the cells were cultured. Next, 100 µL of CellTiter-Glo® reagent was added to each well, either containing cells or ATP standards. The plate was gently shaken for 2 min to ensure mixing, followed by a 10-min incubation at room temperature to allow for the stabilization of the luminescent signal. Luminescence was measured using the Infinite 200 Pro plate reader (Tecan).

### Comet assay

The alkaline comet assay was performed according to the manufacturer’s instructions with minor modifications. Following a 72-h treatment with olaparib (0, 1, 3, 10, 30 μM) or vehicle, cells were harvested, washed in DPBS, and mixed at a 1:10 ratio with pre-melted agarose, cooled to 37 °C. A 50 µL aliquot of agarose containing 2 × 104 cells was pipetted onto CometSlides in technical duplicates. The slides were incubated in the dark at 4 °C for 30 min to allow the agarose to solidify. Subsequently, the slides were immersed in lysis buffer for 60 min at 4 °C, followed by a 30-min incubation in alkaline unwinding solution (0.4 g NaOH, 250 µL 200 mM EDTA pH 10, 49.75 mL dH₂O). Alkaline electrophoresis was performed under cold conditions using the CometAssay Electrophoresis System II (R&D Systems) in an electrophoresis buffer (pH >13). After electrophoresis, the slides were washed twice in dH₂O and once in 70% ethanol, then dried at 37 °C. The slides were then incubated with SYBR Gold (1:10,000) for 10 min at room temperature in the dark. Following incubation, slides were washed three times in dH₂O and dried again at 37 °C. Comet images were captured using the Citation5 imaging system. A total of 50 comets per technical replicate (100 comets per condition across 3 independent biological replicates) were analyzed using CometScore™ software.

### Apoptosis/necrosis analysis by flow cytometry

After 72 h of treatment with olaparib (0, 10, 30 μM) or vehicle, 10^5^ cells/per well were transferred in triplicates to V-bottom 96/well plates. Cells were centrifuged at 2500 rpm for 1 min. The medium was discarded, and the cells were washed twice using Annexin V binding buffer (BioLegend, San Diego, CA, USA) followed by centrifugation. Cells were stained for 15 min at room temperature in the dark with a mixture of antibodies including Annexin V (1:100) and propidium iodide (PI) (1:100), or incubated with Annexin V binding buffer as an unstained, negative control. Next, 150 µL Annexin V binding buffer was added to each well. Cells from triplicates were pooled together into a flow cytometry tube and the signal was measured by Flow LSRFortessa™ Cell Analyzer (BD Bioscience). Flow cytometry data were analyzed using the FlowJo 10.10 software.

### Measurement of cellular bioenergetics

Cellular bioenergetics were measured using the Seahorse XF24 Analyzer after 72 h incubation with olaparib (0, 10, 30 µM) or vehicle, in non-senescent and senescent cells. On the day of the experiment, the cell medium was replaced with bicarbonate-free, low-buffered assay medium (glucose 1 g/l, GlutaMAX 2 mM, sodium pyruvate 1 mM, pH adjusted with NaOH) and the cells were incubated for 1 h at 37 °C in the absence of CO_2_. To perform the Mitostress assay, the appropriate ports were loaded with oligomycin (10 μM), FCCP (3 μM) and rotenone/antimycin A (20 μM each). After measurement of oxygen consumption rate (OCR), the following parameters were calculated: non-mitochondrial respiration (minimum OCR after rotenone/antimycin A injection); basal respiration (the value of OCR before the 1^st^ injection), ATP-linked respiration (the difference between OCR before and after oligomycin injection) and maximal respiration (the difference between OCR after FCCP and rotenone/antimycin A injection). Data were calculated from at least 3 technical replicates of 3 independent biological experiments.

### Statistical analysis

Data are presented as mean ± SEM of at least three biological experiments, with a minimum of two technical replicates. Student’s *t*-test was performed to compare two groups. For multiple comparisons, two-way ANOVA followed by Tukey’s post hoc analysis was used. Differences were considered statistically significant at *p* < 0.05. Statistical analysis was performed using GraphPad Prism 10.2 (GraphPad Software Inc., San Diego, CA, USA).

## Results

### Senescent cells exhibit a marked increase in PARylation and PARP-1 expression

**Fig. 1:**
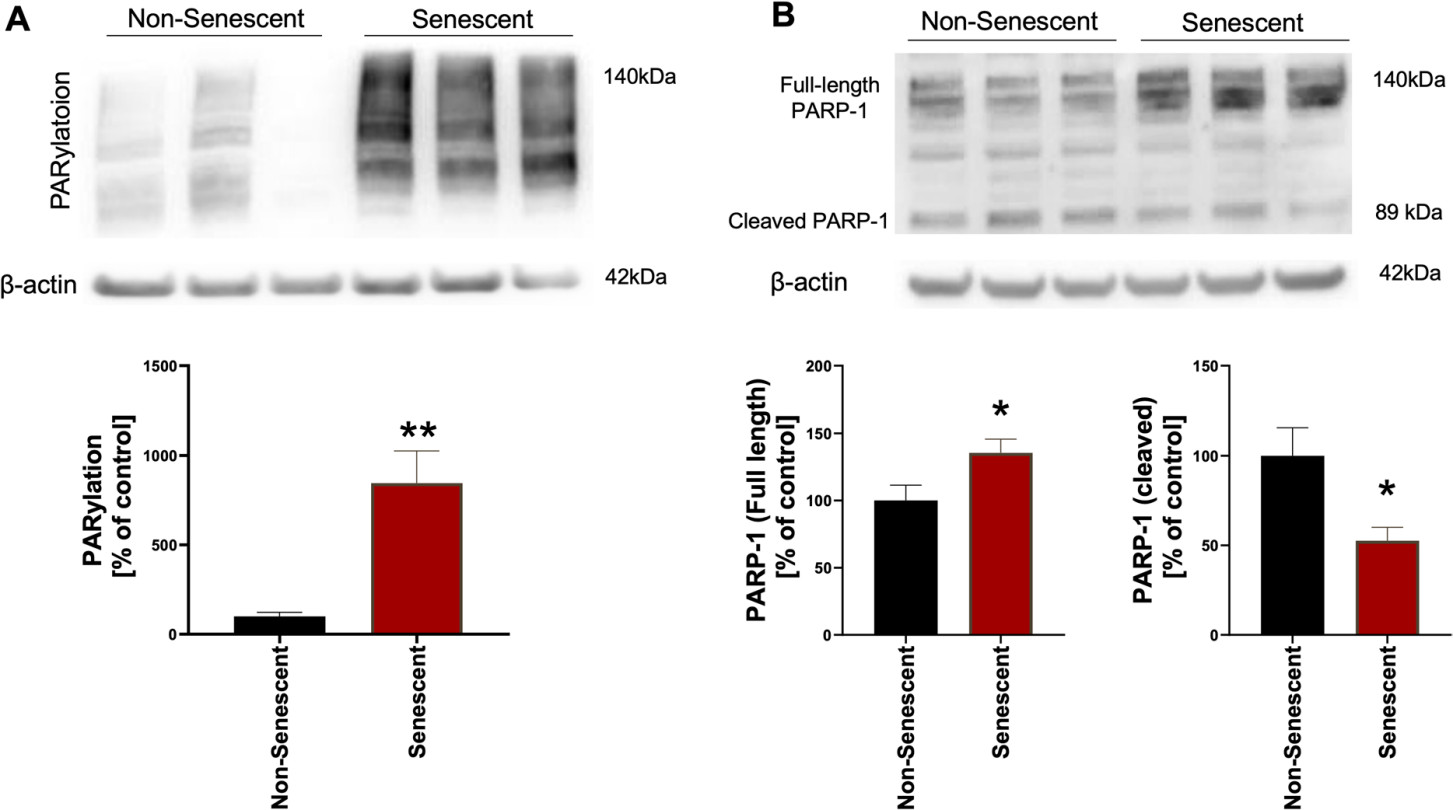
PARylation and PARP-1 expression in non-senescent and senescent RAW 264.7 cells. Representative Western blots and quantitative analyses of protein PARylation (**A**) and PARP-1 expression (**B**), including full-length (140 kDa) and cleaved (89 kDa) forms, in non-senescent and senescent RAW 264.7 cells. Quantitative analyses were shown as mean ± SEM from 6 independent experiments. Symbols * and ** indicate statistical significance at *p* < 0.05 and *p* < 0.01, respectively

In senescent RAW 264.7 cells, the PARylation signal was approximately 8.5 times higher than in non-senescent control cells **(**Fig. 1A**)**. Senescent cells also contained 50% more full-length PARP1 enzyme than non-senescent control cells. Moreover, senescent cells contained approximately 50% less of the inactive, cleaved form of PARP1 than non-senescent control cells **(**Fig. 1B**).**

### Olaparib upregulates various senescence markers

**Fig. 2:**
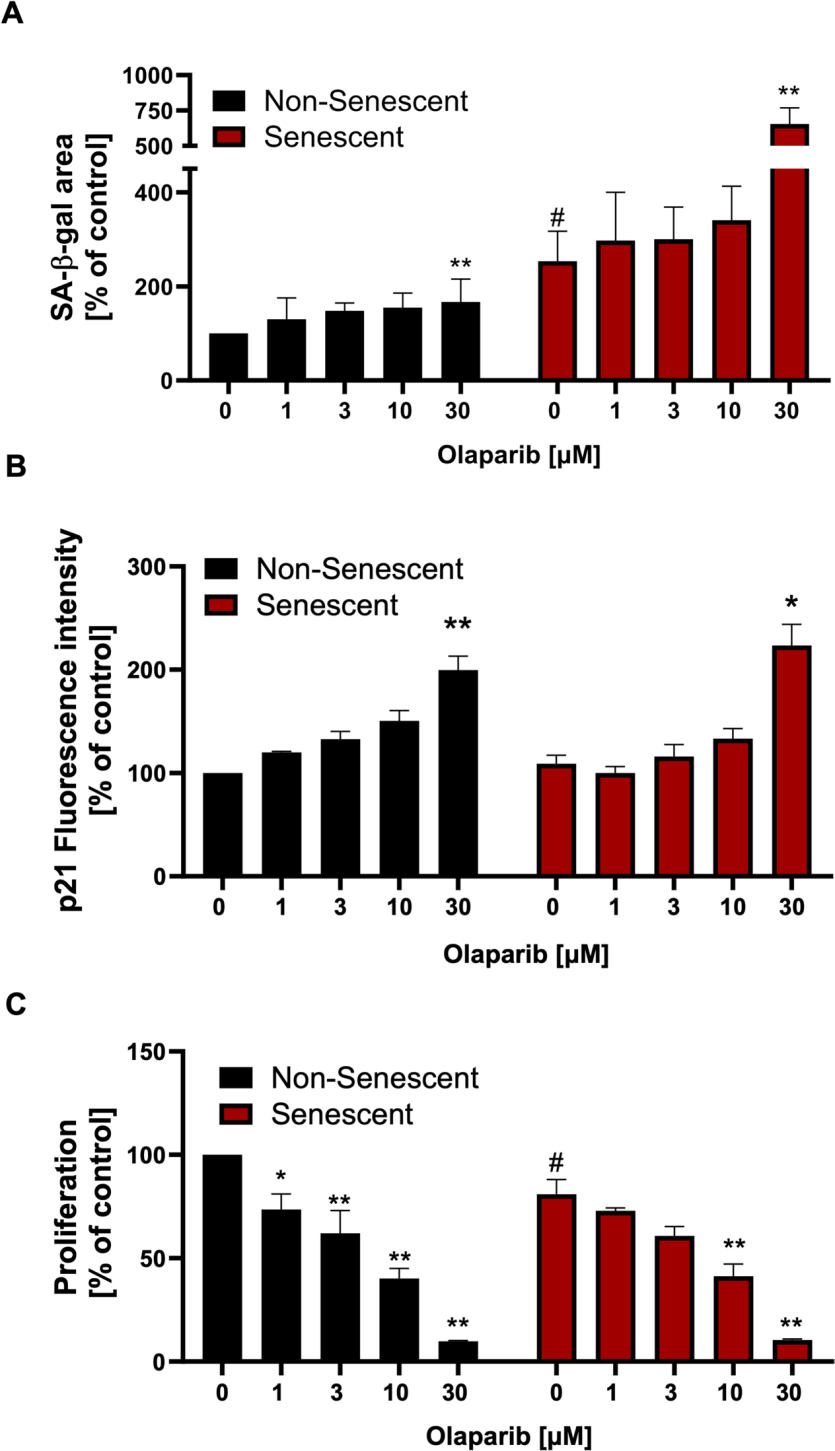
Effect of olaparib on senescence markers in non-senescent and senescent RAW 264.7 cells. Representative images and quantitative analyses of SA-β-galactosidase activity (**A**), p21 expression (**B**), and proliferation rate (**C**) are shown in non-senescent and senescent RAW 264.7 cells in response to 72 h of incubation with olaparib at concentrations of 0, 1, 3, 10, or 30 μM. Quantitative analyses were shown as mean ± SEM from 3 independent experiments. Symbols * and ** indicate statistical significance at *p* < 0.05 and *p* <0.01 respectively. Symbol # indicates statistical significance at *p* < 0.05 between non-senescent and senescent untreated control cells

As expected, SA-β-gal activity was significantly higher in senescent than in non-senescent control cells. Olaparib at 1–10 µM modestly increased SA-β-gal activity in non-senescent control cells, with a significant ~50% increase observed at 30 µM **(**Fig. 2A**)**. In contrast, in senescent cells, 1–10 µM olaparib only had a slight effect on SA-β-gal activity, while 30 µM induced a 2.5-fold increase **(**Fig. 2A**)**.

The expression of p21 was approximately 15% higher in senescent cells compared to non-senescent control cells under baseline conditions. Olaparib exerted a more pronounced effect on p21 expression at 3 and 10 µM in control cells, while both cell types showed a doubling at 30 µM of the PARP inhibitor **(**Fig. 2B**)**.

Olaparib treatment caused a concentration-dependent reduction in proliferation in both cell types: in control cells, the inhibition amounted to approximately 25%, 38%, 60%, and 90% at concentrations of 1, 3, 10, and 30 μM, respectively **(**Fig. 2C**)**, while in senescent cells, the corresponding inhibition values were 10%, 24%, 49%, and 87%, respectively **(**Fig. 2C**)**.

### Olaparib decreases viability and induces cell death in senescent and non-senescent cells

**Fig. 3:**
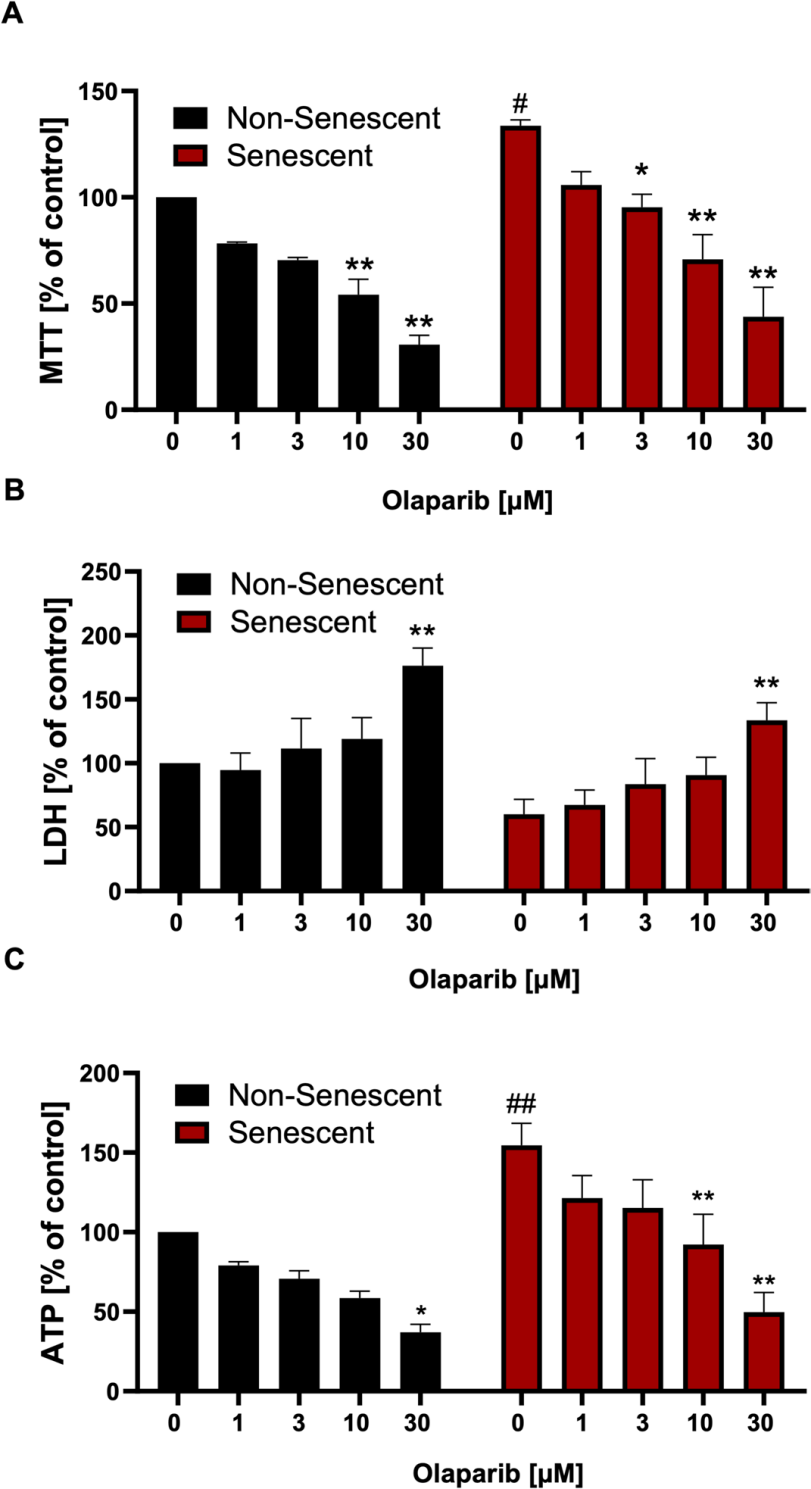
Effect of olaparib on cell viability in non-senescent and senescent RAW 264.7 cells. MTT (**A**), LDH (**B**), and ATP (**C**) assays in non-senescent and senescent RAW 264.7 cells are shown in response to 72 h of incubation with olaparib at concentrations of 0, 1, 3, 10, or 30 μM. Quantitative analyses were shown as mean ± SEM from 3 independent experiments. Symbols * and ** indicate statistical significance at *p* < 0.05 and *p* <0.01, respectively. Symbols # and ## indicate statistical significance at *p* < 0.05 and *p* < 0.01, respectively, between non-senescent and senescent untreated control cells

The MTT assay indicated higher baseline mitochondrial activity in senescent cells than in non-senescent control cells **(**Fig. 3A**)**. Olaparib produced a concentration-dependent inhibition of metabolic activity, with the most pronounced cytotoxic effects observed at 10 and 30 μM in both non-senescent and senescent cells **(**Fig. 3A**)**.

Basal LDH content in the culture medium tended to be lower in senescent cells **(**Fig. 3B**)**. Olaparib induced a concentration-dependent increase in LDH release; at 30 µM, a ~2-fold rise was observed in both cell types **(**Fig. 3B**)**.

In line with the higher mitochondrial activity, ATP levels in senescent cells were 50% higher than in non-senescent control cells under baseline conditions **(**Fig. 3C**)**. ATP production was inhibited by olaparib both in senescent and non-senescent cells in a concentration-dependent manner **(**Fig. 3C**)**.

**Fig. 4:**
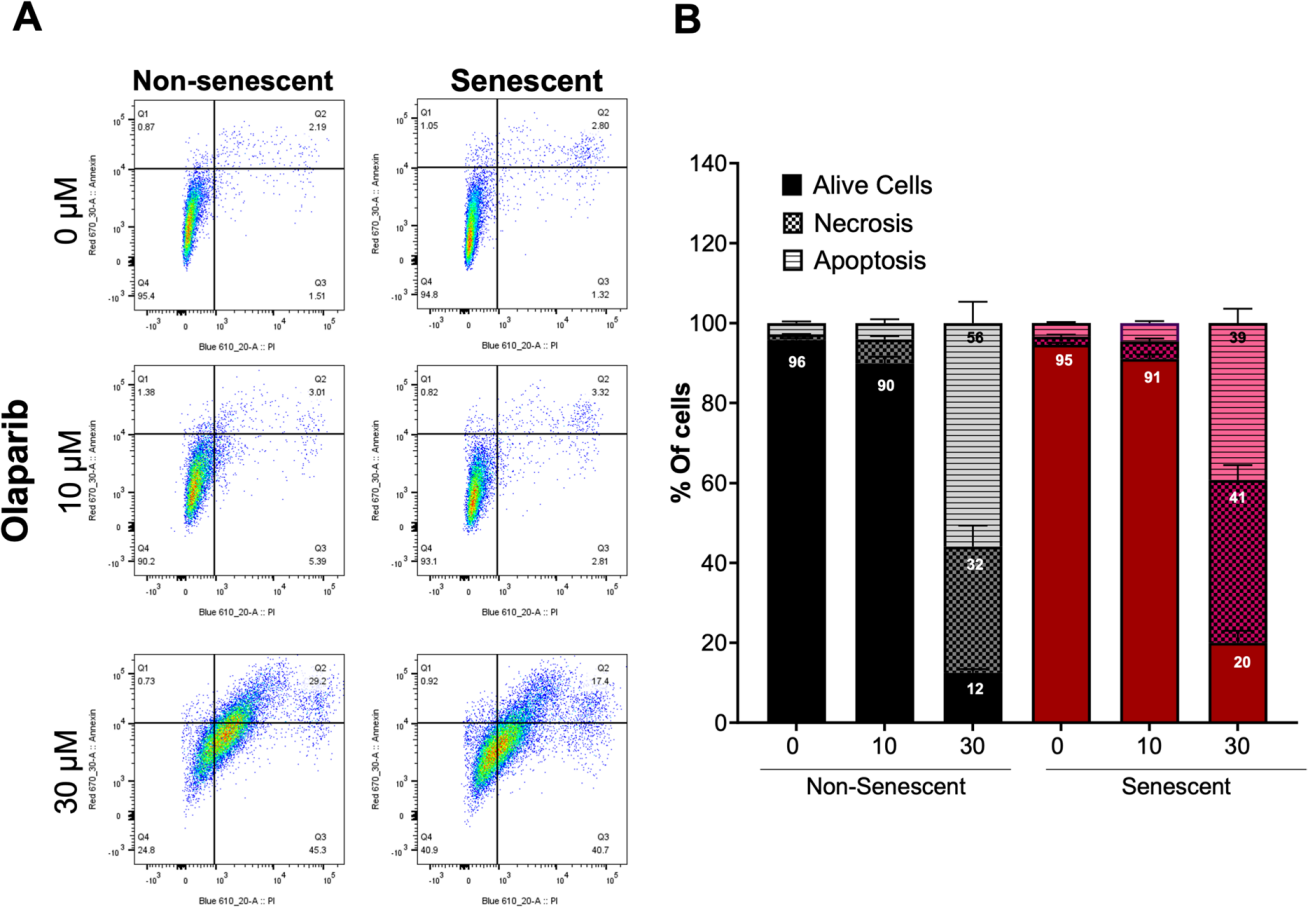
Effect of olaparib on induction of apoptosis and necrosis in non-senescent and senescent RAW 264.7 cells. Apoptosis/necrosis FACS analysis was performed with Annexin V and PI double staining. Section (**A**) shows representative flow cytometry plots of non-senescent and senescent RAW 264.7 cells incubated for 72 h with olaparib at 0, 10, or 30 µM. The percentage of apoptotic and necrotic (**B**) cells was defined based on Annexin V and PI double fluorescence intensity. Quantitative analyses were shown as mean ± SEM from 6 independent experiments. The symbol ** indicates statistical significance at *p* < 0.01

Flow cytometric analysis using double staining for Annexin V and PI revealed that olaparib induces a mixed form of cell death by increasing both the apoptotic and necrotic cell populations **(**Fig. 4A**)**. In non-senescent control cells, olaparib predominantly induced apoptotic cell death (56% vs. 32%), while in senescent cells, 30 µM olaparib led to a greater proportion of necrotic than apoptotic cells (41% vs. 39%) **(**Fig. 4B**)**.

### Olaparib suppresses mitochondrial function and cellular bioenergetics

**Fig. 5.**
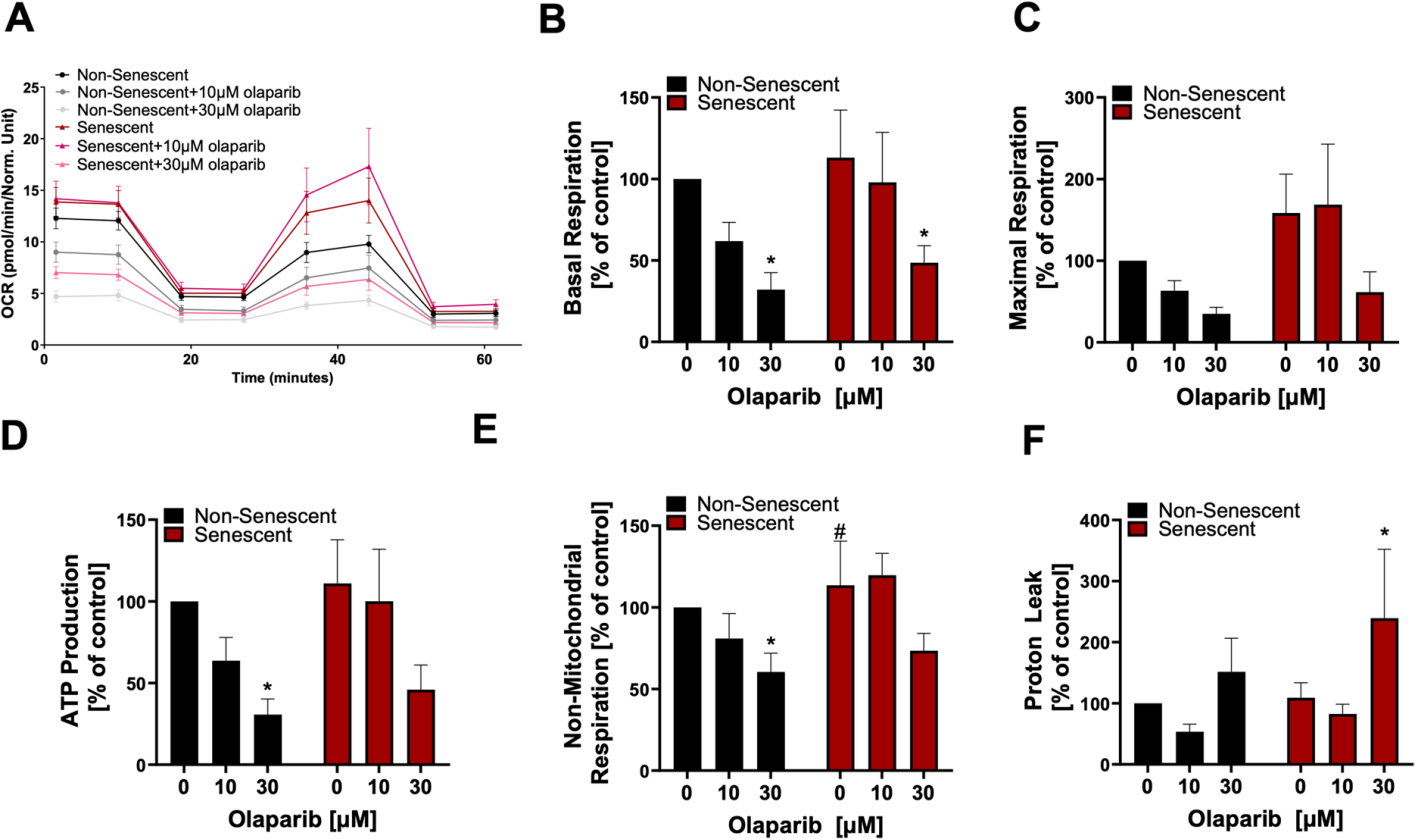
Effect of olaparib on mitochondrial function in non-senescent and senescent RAW 264.7 cells. Oxygen consumption rate (OCR) measurements using Extracellular Flux technology (**A**) after 72 h of incubation of non-senescent and senescent RAW 264.7 cells with olaparib at concentrations of 0, 10, or 30 μM. Basal respiration (**B**), maximal respiration (**C**), ATP production (**D**), non-mitochondrial respiration (**E**) and proton leak (**F**) are shown. Quantitative analyses were shown as mean ± SEM from 6 independent experiments. Symbols * and ** indicate statistical significance at *p* < 0.05 and *p* <0.01, respectively. Symbol # indicates statistical significance at *p* < 0.05 between non-senescent and senescent untreated control cells

In agreement with the findings of the MTT assay, Extracellular Flux Analysis using Seahorse indicated higher baseline mitochondrial activity in senescent cells compared to non-senescent control cells **(**Fig. 5A**)**. Basal respiration **(**Fig. 5B**)**, maximal respiration **(**Fig. 5C**)** and ATP production **(**Fig. 5D**)** all tended to be higher in senescent than in non-senescent cells. Incubation with 10 μM olaparib did not induce significant changes in any of the measured mitochondrial parameters, whereas 30 µM significantly suppressed mitochondrial function in both cell types (Fig. 5).

### Olaparib induces and potentiates DNA damage in senescent and non-senescent cells

**Fig. 6:**
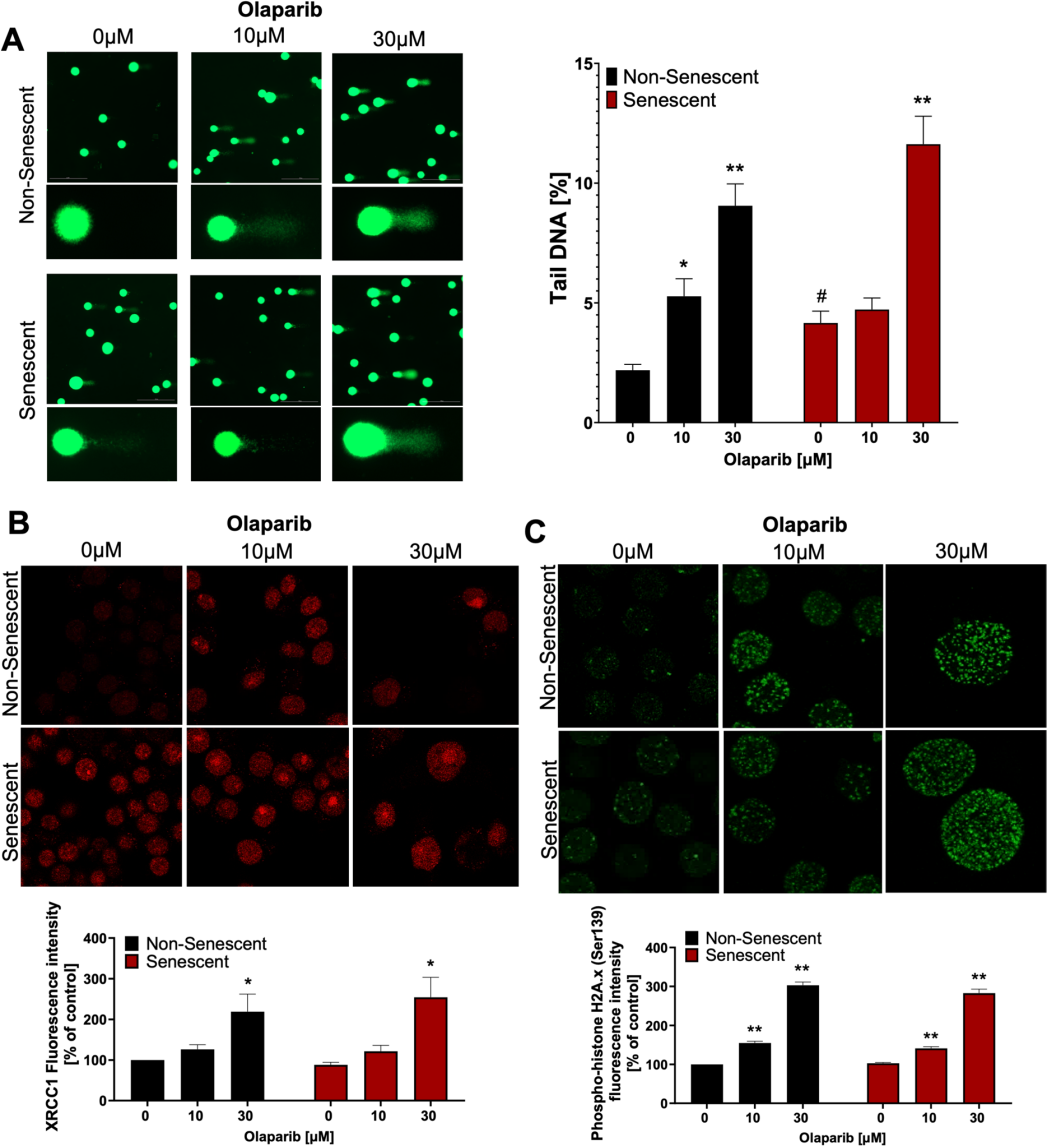
Effect of olaparib on DNA damage in non-senescent and senescent RAW 264.7 cells. Representative images and quantitative analysis of alkaline comet assay (**A**), XRCC1 expression (**B**) and phospho-histone H2 A.X (Ser139) (**C**) are shown following 72 h of incubation is shown in non-senescent and senescent cells following 72 h of olaparib treatment at 0, 10, or 30 µM. Quantitative analyses are shown as mean ± SEM from 6 independent experiments. Symbols * and ** indicate statistical significance at *p* < 0.05 and *p* <0.01, respectively. Symbol # indicates statistical significance at *p* < 0.05 between non-senescent and senescent untreated control

Next, the alkaline comet assay was used to assess the direct effects of PARP inhibition on DNA damage, primarily reflecting double-stranded DNA breakage. Representative images (Fig. 6A) show that non-senescent control cells displayed minimal comet tails, indicating low DNA damage. In contrast, senescent cells showed prominent comet tails under baseline conditions, indicating elevated baseline DNA damage. Quantification confirmed ~2-fold more DNA in comet tails of senescent cells versus non-senescent control cells (Fig. 6A). Treatment with 10 µM olaparib significantly increased DNA damage in non-senescent cells, but had a minimal effect in senescent cells. However, treatment with 30-μM olaparib induced a marked increase in DNA content in comet tails, which was approximately 2-fold higher in non-senescent cells and nearly 3-fold higher in senescent cells, compared to their respective baseline control (Fig. 6A).

XRCC1 expression is a characteristic marker of the cellular response to single-strand DNA breaks. In untreated conditions, XRCC1 fluorescence intensity was ~7% higher in senescent cells compared to controls. Incubation with 10-μM olaparib did not affect XRCC1 expression, while at 30 µM the PARP inhibitor selectively increased XRCC1 expression in senescent cells **(**Fig. 6B**)**.

Cellular response to DNA double-strand breaks was also assessed, by measuring the fluorescence intensity of phospho-H2 A.X in the nuclei. Baseline H2 A.X phosphorylation was marginally higher in senescent cells, and olaparib treatment elevated it similarly in both groups (Fig. 6C).

## Discussion

Senescent cells arise during aging and are characterized by functional and morphological dysfunctions, including permanent cell cycle arrest, the senescence-associated secretory phenotype, and telomere shortening [25]. Cellular senescence is a highly conserved cellular program triggered in response to a wide variety of stressors, including telomere shortening, oncogene activation, oxidative stress, and DNA damage. Once initiated, senescence results in irreversible growth arrest and is associated with chromatin remodeling, metabolic reprogramming, elevated SA-β-gal activity, and secretion of pro-inflammatory factors (SASP) [26, 27]. While senescence originally evolved as a tumor-suppressive mechanism to prevent propagation of damaged cells, its accumulation over time, particularly in post-mitotic tissues and stem cell niches, contributes to age-associated tissue dysfunction, chronic inflammation, and the pathogenesis of degenerative diseases [25–27].

A substantial body of literature demonstrates the importance of DNA damage during the aging process [28–30]. Since PARylation reactions and PARPs –– most significantly, PARP1 –– are involved in DNA damage repair [1–4], our working hypothesis was that by inhibiting PARP activity, the process of cellular senescence may become accelerated. We wished to determine if such an effect may be different, perhaps more pronounced, in high-passage (senescent) cells than in low-passage (non-senescent) control cells. Among various tissue types, immune cells are particularly susceptible to senescence, a process termed *immunosenescence*. This phenomenon encompasses a range of functional impairments, including reduced phagocytosis, impaired antigen presentation, and altered cytokine secretion, which compromise host defense and contribute to chronic inflammation in aging tissues [31]. Macrophages, as central regulators of innate immunity, undergo functional decline during aging and are also involved in clearing senescent cells; paradoxically, their own senescence may further aggravate the pro-inflammatory milieu [32]. Immunosenescence has been implicated both in physiological aging and in the context of antitumor responses [33, 34]. Thus, we selected a macrophage cell line for our current investigation.

We have compared the effects of the PARP inhibitor olaparib in senescent and non-senescent macrophages. Poly(ADP-ribose) polymerases (PARPs), especially PARP-1, are crucial enzymes involved in DNA damage sensing and repair. Upon activation by DNA strand breaks, PARP-1 catalyzes the addition of poly(ADP-ribose) (PAR) chains to nuclear proteins, thereby facilitating chromatin relaxation and recruitment of DNA repair machinery [1–4]. However, excessive PARP activation can lead to NAD^+^ depletion and bioenergetic crisis, contributing to cell death in various pathophysiological conditions [3, 4].

Olaparib is a potent PARP-1 and PARP-2 inhibitor widely used in oncology, particularly in BRCA-mutated cancers where impaired homologous recombination repair creates a synthetic lethality context [4]. However, it is increasingly recognized that olaparib also inhibits other members of the PARP family (e.g., PARP-3, PARP-4, PARP-5a/b) at higher concentrations, and may interact with off-target proteins, contributing to cytotoxic effects beyond its canonical mechanism of action [35–40]. These off-target effects may become especially relevant when considering the broader physiological implications of PARP inhibition, such as effects on mitochondrial function, inflammation, or cellular senescence.

The first observation of our study was that senescent murine macrophages exhibit a marked increase in the PARylation of multiple proteins, compared to non-senescent control cells. This observation may be, in part, related to the upregulation of PARP1 protein itself, but more likely it represents a response to the higher degree of baseline DNA damage that was also noted in the senescent cells in the Comet assays in our experiments. Interestingly, in senescent cells the relative proportion of cleaved PARP was lower than in non-senescent controls. This finding is consistent with the observed higher PARylation in the senescent cells (since cleaved PARP1 is inactive), but does not align with the expectation that a higher oxidative stress burden and pro-inflammatory activation in senescent cells would lead to increased, rather than decreased, PARP1 cleavage. This seemingly paradoxical decrease in cleaved PARP1 be, however, consistent with the relative resistance of senescent cells to apoptosis (caspase-mediated PARP cleavage).

Our current working hypothesis is that senescent cells have a higher degree of DNA damage, at least in part due to increased oxidative/nitrative stress (and perhaps, in part, due to various alterations in their DNA repair processes). As a result, a more pronounced PARP activation ensues, as a direct consequence of the more severe baseline DNA damage in senescent cells. Indeed, prior studies have also observed higher levels of oxidant generation and decreased DNA integrity in senescent cells [41–45]. However, the question as to whether senescence or physiological aging is associated with increased or decreased PARP expression or PARylation is less clear and is likely to be dependent on the model of senescence used; in some models, senescence was reported to increase PARP1 expression and activity, while in others, decreases were reported [46–53]. Moreover, overactivation of PARP-1 was also previously observed in various age-associated diseases as well as in a variety of diseases associated with oxidative stress and inflammation, which are key factors in the aging process [4, 11, 54–56].

**Fig. 7:**
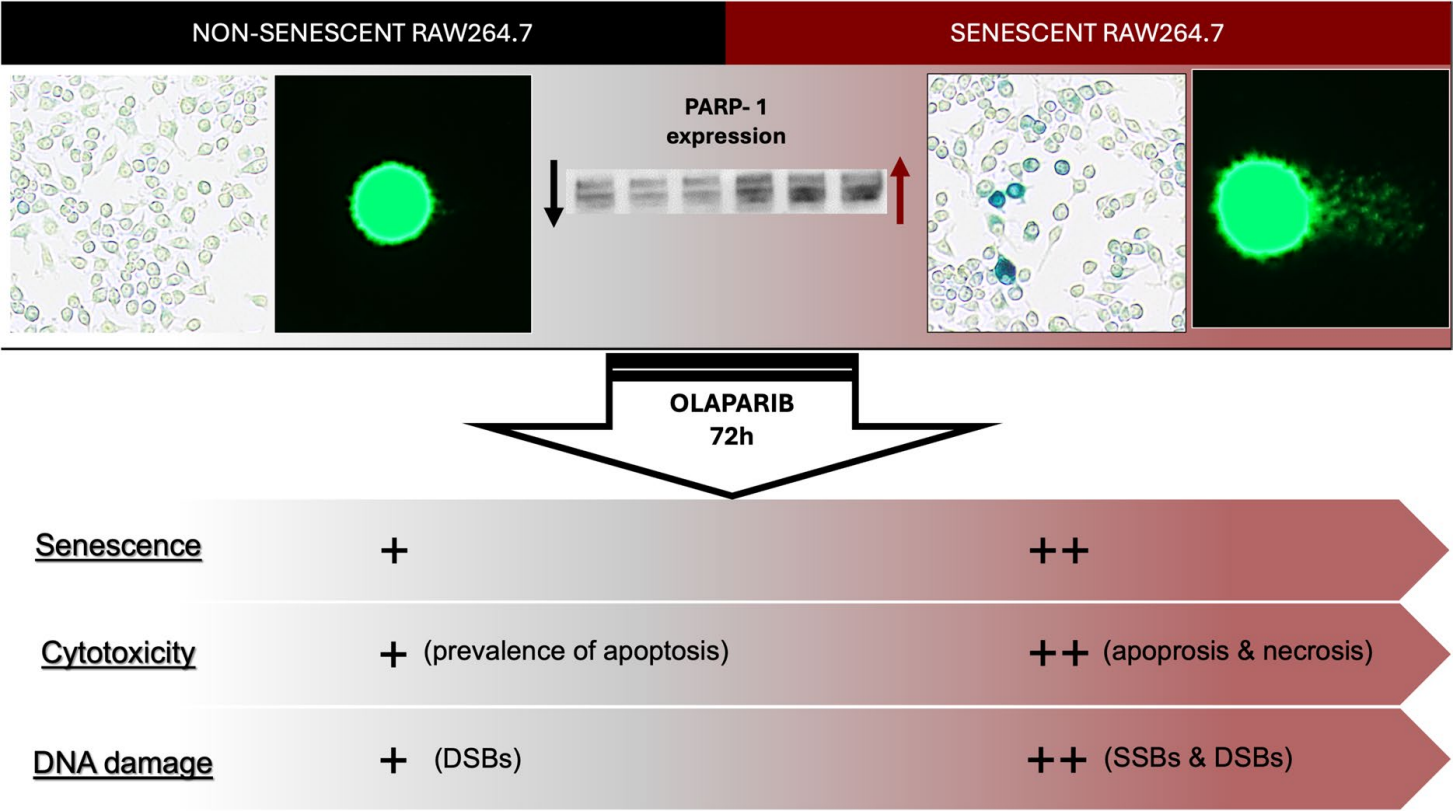
Summary: enhanced sensitivity of senescent cells to olaparib-induced cytotoxicity suggests the potential for olaparib to serve as a selective eliminator of senescent immune cells. SSBs: single strand breaks; DSBs: double strand breaks

Our study reveals that olaparib exerts markedly different effects in senescent versus non-senescent macrophages. Treatment with olaparib further exacerbated DNA damage in both cell types but the increases in senescence-associated markers (e.g., SA-β-galactosidase activity, p21) and cytotoxicity were more pronounced in senescent cells than in non-senescent controls **(**Fig. 7**)**. Notably, at 30 µM, olaparib led to predominantly necrotic cell death in senescent cells, while apoptosis was the dominant form in non-senescent cells. This differential vulnerability likely stems from the combination of higher baseline damage and a blunted capacity for further DNA repair in senescent cells, tipping them toward a necrotic pathway of cell death after pharmacological inhibition of PARP activity.

Olaparib is a highly potent inhibitor, with an IC_50_ of approximately 10 nM against human recombinant PARP1; however, it also inhibits other PARPs with variable potencies (ranging from 10 nM to 10 µM) and exhibits a ‘trapping effect’ on PARP1 at replication forks [57]. Even in cell-based systems, the detectable PARylation response is completely or near-completely inhibited at low (1–3−10 µM) concentrations [58–62]. Interestingly, although low doses of olaparib (1–10 µM) abolish PARylation, much higher concentrations (30–60 µM) are often needed for cancer cell killing [60–67]. One explanation is that even minimal residual PARP activity is enough for cell survival, so near-complete inhibition is required. Alternatively, olaparib’s anticancer effect might require inhibiting other PARP isoforms or additional targets, which only occurs at higher concentrations. Indeed, olaparib remains cytotoxic in cancer cells even after PARP1 is silenced, although its IC_50_ shifts to the right (from approximately 10 to approximately 500 µM) [60]. The relevant messages from the above considerations are that (i) the concentrations of olaparib used in the current study (even the highest one, 30 µM) are potentially clinically relevant and (ii) that the effects of olaparib seen at the higher end of the concentration-response curve may not necessarily be related to inhibition of PARP1, but may be related to actions on other PARPs. In the context of the current study, it is conceivable that the effects of olaparib may result from a combination of inhibition of PARP1 and an inhibitory effect on other PARP isoforms. Indeed, a recent study demonstrates that inhibition of PARP2 in the centrosomes exerts a senescence-promoting effect in human breast cancer cells in vitro [68].

One surprising finding was that senescent RAW 264.7 cells exhibited elevated baseline mitochondrial activity and ATP levels, as determined by MTT assay, Seahorse extracellular flux analysis, and luminescence-based ATP quantification. Most review articles associate cellular aging and senescence with metabolic decline, particularly mitochondrial dysfunction and reduced oxidative phosphorylation, and there is a significant body of literature aimed at improving mitochondrial function in aging [25, 27, 42, 45, 69–73]. However, a more careful analysis reveals a more heterogeneous picture, showing in many cases decreased, in some cases unchanged, and in several reports increased cellular bioenergetics in aging/senescence (Table 1) [74–114]. Some of these differences may arise from differences in cell types, senescence models, or measurement methods of bioenergetics. For instance, mitochondria from aged or senescent tissues may be more prone to experimental artifacts during isolation. We regard in situ mitochondrial function assessments (e.g., Seahorse XF analysis) as more physiologically relevant than assays involving isolated mitochondria. There may also be important cell-type differences. For instance, certain senescent phenotypes –– including adipocytes, muscle cells, stem cells and certain cancer cells –– were found to exhibit hypermetabolic states during senescence, in some cases associated with increased mitochondrial biogenesis and/or enhanced glycolysis [96, 101–110, 112, 113]. In the studies where aging/senescence was found to be associated with decreased bioenergetic function, the underlying mechanisms were attributed to oxidative mitochondrial injury, impairment of mitochondrial DNA replication and increased mitochondrial DNA mutations, inhibition and downregulation of various respiratory chain complexes, increased mitochondria uncoupling, and others [69–73]. On the other hand, in the studies where increased bioenergetics was reported in aging/senescence, the underlying mechanisms include metabolic shifts towards glycolysis or for alternative energy substrates such as glutamine or fatty acids [90, 103, 115]; increased mitochondrial mass, possibly, at least in part due to reduced mitophagy or mitochondrial fusion [105, 107]; and a variety of other potential compensatory mechanisms in response to oxidative or nitrative protein and/or DNA damage [96, 101, 108]. These responses — possibly as part of the SASP program or stress adaptation — may reflect cellular efforts to maintain viability under persistent damage.

**Table 1:**
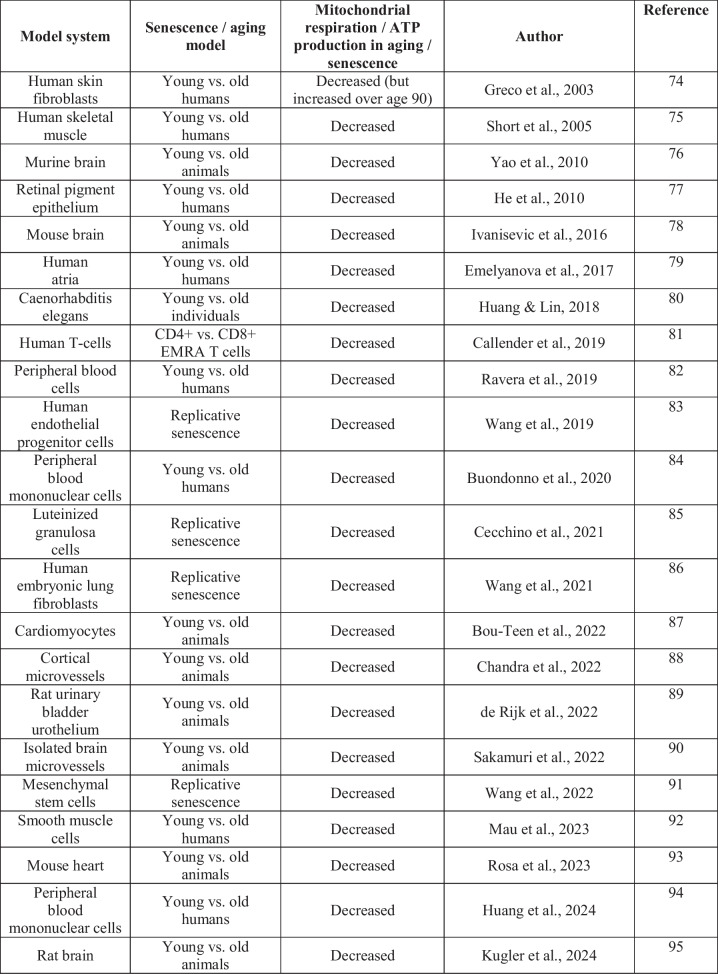

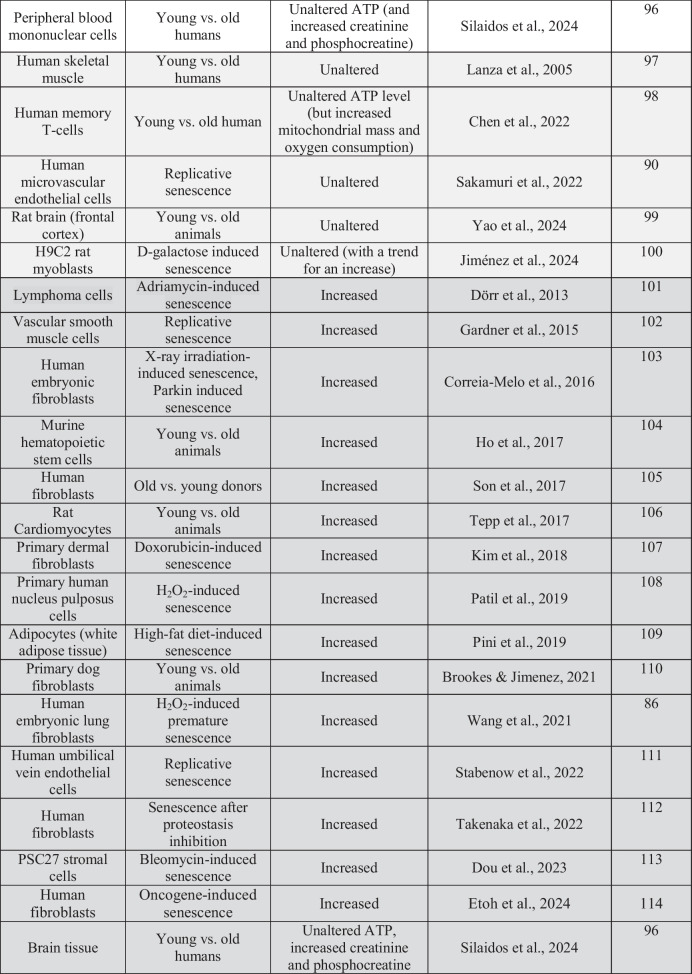
Changes in mitochondrial function in various aging/senescence models

Further studies are needed to clarify the mechanisms behind these divergent findings. In the in the context of our study (DNA strand breakage and PARP activation), our working hypothesis is that the higher bioenergetic activity in senescent cells –– which is reflected in higher mitochondrial activity (i.e. the MTT assay), and mitochondrial oxygen consumption (i.e. Extracellular Flux Analysis), but only reflected to a lesser degree on cellular ATP content –– may reflect a compensatory mechanism, whereby the senescent cells need to mobilize more of their bioenergetic reserve, in order to support the higher demand for various repair processes (e.g., repair of broken DNA strands). In this context –– especially when we consider that the senescent cells in our study are associated with markedly higher nuclear PARylation –– it is important to mention recent studies demonstrating that PAR can be converted in the nucleus to support the bioenergetic need of nuclear DNA repair enzymes [116]. Via this mechanism, a proportion of the bioenergetic ‘currency’ –– which is higher in senescent cells than in non-senescent cells –– may be transferred into the nucleus to support the cells’ increased bioenergetic requirement for DNA repair. Elevated ATP levels may serve as “hydrotopes,” maintaining phase separation and preventing protein aggregation [117, 118] — functions that may be especially critical in senescent cells [119, 120].

The findings reported in the current study carry potential translational implications. Senescent cells accumulate in aging tissues and contribute to age-related decline and chronic inflammation. Recent studies have proposed *senolytic* therapies — agents that selectively eliminate senescent cells — as a strategy to delay aging or enhance health span [121]. Our data suggest that olaparib, particularly at higher concentrations, may exhibit some *senolytic-like* activity by preferentially targeting senescent immune cells, including macrophages. However, these effects are not absolute: olaparib also exerted significant effects on many of the observed parameters in non-senescent cells. Thus, the possibility whether PARP inhibitors might be repurposed or optimized for senescence-targeting therapies remains to be further explored (including the necessary in vivo validation). If such an action were to be confirmed in subsequent studies, it may be beneficial in oncology, where immunosenescence contributes to tumor progression [33, 34]. Conversely, enhanced cytotoxicity toward senescent cells may increase the risk of adverse effects in older patients receiving olaparib: the elevated cytotoxicity toward senescent immune or parenchymal cells could exacerbate immune dysfunction or tissue degeneration.

The current study has several limitations. First of all, of our conclusions are based on experiments using a *murine* system and a *cell line*. Although replicative senescence is commonly investigated in various monocytic, macrophage, lymphocytic and microglial cell lines [122–130], replicative senescence in immune cell lines remains an artificial experimental system. Thus, the question if the findings also apply to *human* and *primary* immune cells remains to be determined in follow-up studies: it will be important to determine if similar pro-senescent or cytotoxic effects of olaparib may also occur in primary immune cells from aged animals or human donors. Translating these findings to primary systems would greatly enhance the clinical relevance of the current report.

In addition, it would also be an interesting follow-up direction to test olaparib in combination with other senolytic agents (e.g., dasatinib, quercetin) to assess potential synergy or cell-type specificity. Such experiments could be particularly interesting given the dual apoptotic/necrotic response profile observed in response to olaparib.

The vast majority of the literature on olaparib and other clinically used PARP inhibitors regarding senescence focuses on the potential senescence-inducing effect of these agents in cancer cells [16–19, 131–134]. The results presented in this report provide novel insights into the differential responses of non-senescent and senescent macrophages to PARP inhibition. Our findings demonstrate: (i) elevated PARP activity and DNA damage in senescent macrophages; **(ii)** enhanced sensitivity to olaparib-induced cytotoxicity; and (iii) the potential of olaparib as a selective senescent cell eliminator. We also observed (iv) an unexpected increase in bioenergetic function in senescent cells. These findings prompt further investigation into the dual roles of PARP inhibitors in aging and cancer, and their potential utility as (immuno)senolytic agents.

## Data Availability

The datasets used and/or analyzed during the current study are available from the corresponding author on reasonable request.

## Abbreviations

PARP: Poly (ADP-ribose) polymerase
BrdU: 5-Bromo-2′-deoxyuridine
DMSO: Dimethyl sulfoxide
DMEM: Dulbecco’s modified Eagle’s medium
MTT: Thiazolyl blue tetrazolium bromide
LDH: Lactate dehydrogenase
SA-β-gal: Senescence-associated β-galactosidase
DPBS: Dulbecco’s phosphate-buffered saline
PI: : Propidium iodide (PI)
